# Morpometric and molecular characterization of Surguli goat through CO1 gene in district Kohat

**DOI:** 10.1080/10495398.2023.2290528

**Published:** 2023-12-23

**Authors:** Muhammad Munir Khan, Syed Muhammad Suhail, Hafiz Abdul Majid, Ijaz Ahmad, Umer Sadique, Rajwali Khan, Iftikhar Ahmad, Asim Ijaz, Khalid Khan, Farhad Ali, Muhammad Saeed Khan, Ahmed A. El-Mansi

**Affiliations:** a Department of Livestock Management, Breeding and Genetics, The University of Agriculture, Peshawar, Pakistan; b Department of Livestock and Dairy Development (Research Wing), Government of Khyber Pakhtunkhwa-Peshawar, Peshawar, Pakistan; c Department of Animal Health, Faculty of Animal Husbandry and Veterinary Sciences, College of Veterinary Sciences, The University of Agriculture, Peshawar, Pakistan; d Biology Department, Faculty of Science, King Khalid University, Abha, Saudi Arabia

**Keywords:** Kohat, Surguli goat, morphometric, phylogenetic, CO1, haplotypes, *Capra ibex*

## Abstract

The present study was designed with the aim to study morphometric characterization as well as phylogeny and diversity of the local Surguli goat at their breeding tract district Kohat through mitochondrial DNA region, i.e., Cytochrome C Oxidase Subunit One (CO1) gene. Morphometric data and blood samples were collected from thirty (30) pure goats. Morphometric analysis showed that sex had significant effect (*p* < 0.05) on body weight, body length, hearth girth and horn length while no significant effect (*p* > 0.05) was observed for other characteristics. The results also indicated that age had significant effect (*p* < 0.05) on height at rump, ear length, horn length and tail length while no significant effect (*p* > 0.05) was observed for other characteristics. The phylogenetic analysis through CO1 nucleotide sequences within nucleotide range 1–767 showed nine polymorphic sites segregating into eight haplotypes. The mean intraspecific diversity and mean interspecific diversity were calculated as 0.23 and 2.36%, respectively. Phylogenetic tree revealed that Capra Ibex and native Surguli goat have common ancestors. The morphometric and molecular results obtained from the present study can be exploited as a selection tool for breeding and overall improvement.

## Introduction

Asia comes first having 597 million of goat population, which is 59% of the total world population. Asian countries’ present share of goat products, such as milk, meat and skin in the world are estimated to be 58.3, 70.7 and 76.5 percent, respectively.^1^

Small ruminants, particularly native breed kinds, play a significant role to the livelihoods of a considerable part of human population in the tropics from socio-economic aspects.^2–4^ Thus, combined trials with emphasis on administration and genetic progress to improve animal outputs are of decisive significance.^5^^,^^6^ Economical and biological efficiency of production enterprises generally improves by increasing productivity and reproductive performance of small ruminants.^7–11^

Pakistani goats are originated from two wild species Markhor (*Capra falconeri*) and Ibex (*Capra ibex*).^12^ Pakistan, as the world’s leading goat producer, contributes 80.3 million goats to the total livestock population of the country. The annual contribution of goat production to the national economy was calculated to be 991 thousand tons of milk and 0.3 million of skin. Furthermore, goat meat is estimated to be 275 tons.^13^

There have been 36 goats breeds identified so far from different geographical zones of Pakistan.^12^ Other breeds can be added to this list because of within breed diversity which is not very well presented and a number of unregistered breeds, especially in Khyber Pakhtunkhwa province. It has a variety of documented native goat breeds and undocumented goats. Surguli goat is one of the undocumented goats belonging to Kohat division and the surrounding areas and can also be found in other southern region i.e., Lakki Marwat and D.I Khan.^14^ The base line survey is not available for Surguli goat; however, research study has been conducted on morphometric characterization in comparison with other goat breeds of the province.^14^

The phenotypic and genetic characteristics of animals are used to characterize them. Coat color, hair or skin, horns (shape and age), adult size in terms of live weight and body measurements such as wither height are often used in phenotypic characterization of a breed Mahammi et al.,^15^ Meghen et al.,^16^ Benhamadi et al.,^17^ Labbaci et al.,^18^ Mediouni et al.,^19^ Djaout et al.,^20^ Benyarou et al.,^21^ Belantar et al.,^22^ Benyarou et al.,^21^ Meghelli et al.^23^ and Mogharbi et al.^24^

Besides from morphometric characterization of breeds, the mitochondrial genome has been regarded nowadays as a strong tool for identifying and determining genetic markers and diversities to explore the phylogenetic associations amongst populations or species. This novel method is used globally due to quick evolving, easiness in sampling and radially obtainable from any tissue.^25–29^ Every eukaryotic cell is enriched with mitochondrial organelle located inside the cytoplasm.

Mitochondria has small sized maternally transmitted genome which is lacking genetic recombination and repair mechanism. Mitochondrial DNA is organized as a circular double stranded DNA molecule in most eukaryotes.^30^ The strands are categorized into H-strand (Heavy strand) and L-strand (Light strand). H-strand is guanine rich as compared to L-strand, which is cytosine rich. The length of mitochondrial DNA varies between species to species (15,000–17,000 bp).

Cytochrome c oxidase subunit one gene (CO1) is one of 37 genes of mitochondrial DNA which codes for protein and is located from nucleotide 5904–7444 on H-strand.^31^ The CO1 gene is pronounced globally for the evaluation of molecular diversity as it proved a powerful toolkit for identity of closely related species.^32^ Research studies have confirmed that CO1 mitochondrial gene is extremely conservative across species.^33^^,^^34^ The CO1 gene has successfully differentiated various ruminant species into different taxa and has been proven to be a reliable tool for ruminant phylogeny and taxonomy.^35^ Through CO1 gene sequences, the domestic Pakistani goat breeds can be differentiated from the foreign breeds.^36^ Conservation management and specie monitoring need utilization of the statistics attained through genetic methods like phylogenetics, genetic information and evolution.^37^ In recent years, studies have revealed the relationships between Pakistani goat breeds and species,^12^ therefore the present study was aimed to characterize the Surguli goat based on morphometric characterization as well as molecular characterization through CO1. This will be the first study of its kind on phylogeny and identification of Khyber Pakhtunkhwa goat breeds through CO1 gene. The key goal of the present study was to examine the origin of Surguli goat and level of the genetic diversity within this goat for future documentation and considering Surguli an independent goat breed.

## Materials and methods

### Sample collection and DNA extraction

**Figure 1.**
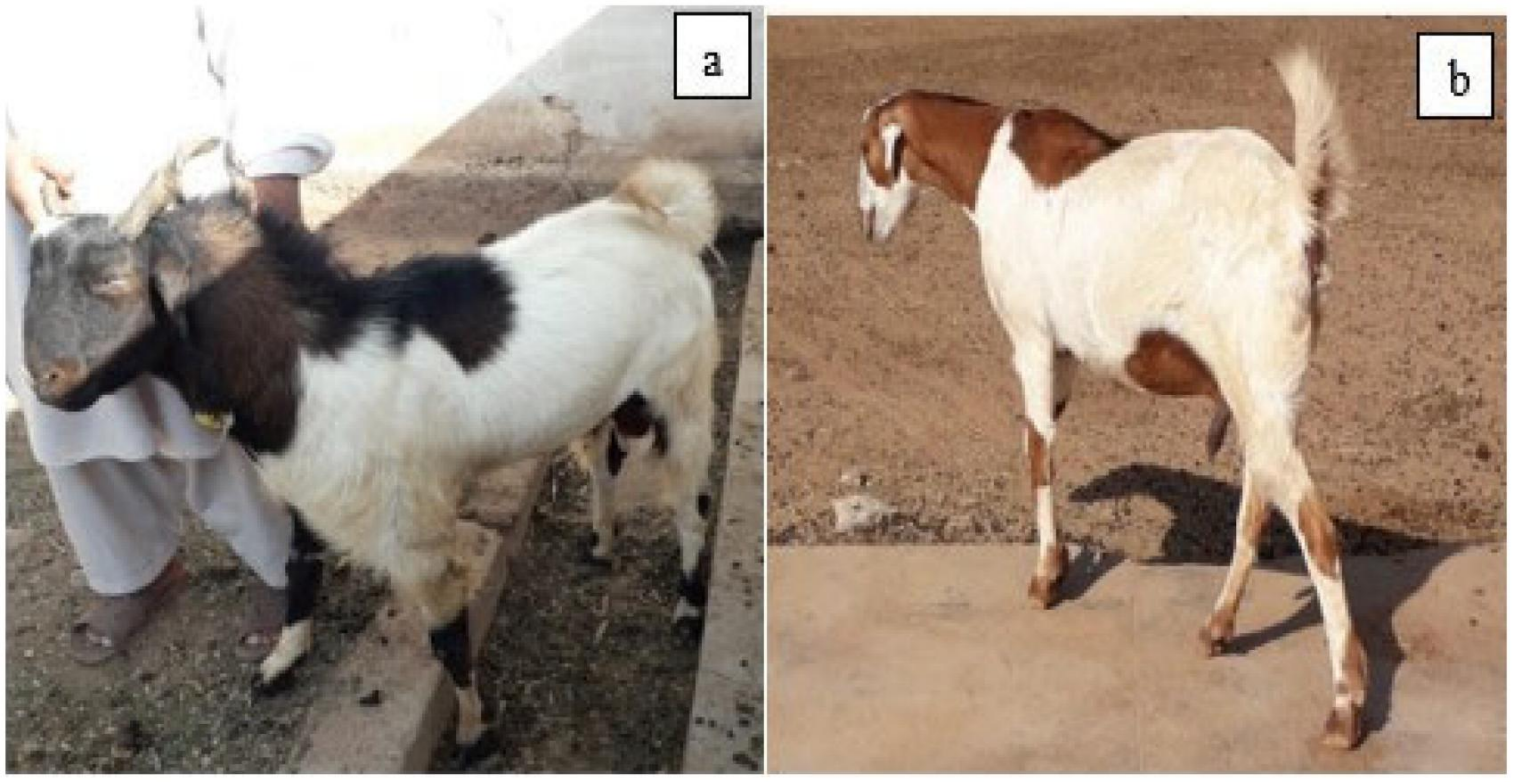
(a) Surguli buck, (b) Surguli doe.

The breeding track of Surguli goat district Kohat located at the Southern belt of Khyber Pakhtunkhwa province was visited in April 2021 and true representative goats were selected for morphometric and molecular characterization. The blood samples and morphometric data of Surguli goats were collected at Arid Zone Small Ruminant Research Institute, Ghulam Banda, Kohat, Pakistan including registered farmers of the area. A team of experts from Arid Zone SRRI Ghulam Banda, Kohat (33.5889° N, 71.4429° E) was accompanied to select a typical Surguli goats as shown in the Fig. 1. Moreover, history-based pedigree was recorded from the owner of each animal to ensure pure breed. A total of thirty Surguli goats including fifteen(3 bucks and 12 does) from research farm and fifteen (6 bucks and 9 does) from registered farmers of different age groups i.e., 2–5 years of age having no common ancestors were randomly sampled for morphometric data and blood collection.

An electronic weighing balance (JMS Machineries Pvt. LTD.PAK) and a measuring tape (cm) (JMS Machineries Pvt. LTD.PAK) were used to measure the body weight (kg) and other morphometric characteristics, respectively.

Morphometric parameters of the Surguli goats that were collected included body weight, body length, hearth girth, horn length, ear length, head length, neck length, height of animal at wither and rump and tail length.

Animal handling, restraining and collection of data were performed in accordance with the descriptions lists provided by FAO.^38^ Morphometric data were collected on semi-structured data collection sheets (Annexure 1) along with visual judgment and measurement. It was made sure that animals were standing on a plan surface during their body measurements.

For molecular characterization blood sample were collected from the animals, which were characterized morphometrically. The surface of jugular vein was disinfected with 70% ethanol and punctured with sterile and disposable syringe for collection of 3 mL blood, which were gently transferred to EDTA tubes (REF-XLGA-E3K3, Xinle®, China). DNA was extracted through non-enzymatic salting out method from blood samples as described by Suguna et al.^39^ The research work was performed at Histopathology Lab, College of Veterinary Sciences FAH & VS, The University of Agriculture Peshawar, Khyber Pakhtunkhwa, Pakistan (34.0206° N, 71.4814° E).

### PCR amplification of CO1

The specific region of 767-bp of *CO1* gene was amplified using: F-5′-ACAGGACTTGGTAAAAAGAGG-3′ and R-3′-ATACTTCAGGGTGTCCAAAG-5′ primers (accession no. KP662714.1). The designed primers were sent to Macrogen®, Korea, for synthesis. A total of 25-µL PCR reaction was prepared for each sample in a PCR tube by adding 5 µL of master mix (Cat. No.SM213-0250, GeneDireX, Inc.), 1.5 µl of reverse primer and 1.5 µL of forward primer, 12 µl of PCR water and 5 µL of template DNA. The reaction was carried out in thermal cycler BIORAD^®^ using the following protocol: 94 °C for 5 min and 34 cycles of 94 °C for 30 s, 55.1 °C and 59 °C for 30 s, 72 °C for 1 min and 72 °C for 10 min.

### Sequencing of CO1 gene

The targeted region on isolated DNA was amplified by PCR techniques using specific primers of CO1 gene. The sequencing reactions of mitochondrial CO1 gene were performed through Sanger sequencing method, as described by Sanger et al.^40^ The targeted regions were subjected to chain termination PCR and million to billion copies were terminated at random lengths by 5′-ddNTPs. Then, terminated oligonucleotides were separated in gel electrophoresis via supply of electric current. Finally, gel was analyzed, and DNA sequence was determined by fluorescence tags through automated Sanger sequencing and results were generated by computer in AB1 format.

## Data analysis

### Morphometric analysis

The morphometric data was organized in Microsoft excel sheet and analyzed using statistical package SAS, version 6.9. Body weight, which is economic trait were taken as response variable. Keeping in view the importance of body weight as an important growth parameter, the same was also predicted from body length and hearth girth as class variables. Following regression model was used:

$$Y=\beta_{0}+\beta_{1}x+\varepsilon$$

Where:

Y = The dependent variable (body weight)

β_0_= Intercept

β_1_=Independent variables (Body length and hearth girth)

ε= random residuals

To find the statistical differences among the sample goat populations, a general linear model approach (PROC GLM) was applied. Sex and age groups of the goats were fitted as fixed variables whereas body weight and other morphometric parameters as response variables, least square means with their corresponding standard errors were found out for each parameter over sex and age to test statistically difference by Duncan Multiple Range test. The following general linear model was used.

$$Y_{\text{ijkl}}=\mu +{\text{ A}}_{i}+{\text{ S}}_{j}+{\text{ e}}_{\text{ijk}}$$

Where;

Y_ijkl =_ each dependent variable

µ = Population mean

A_i_ = i^th^ effect of age (2, 3, 4 and 5 years)

S_j_ = j^th^ effect of sex (Male and Female)

e_ijk_ = random residuals error

### Molecular analysis

The reference sequence of COI gene (accession no. KP662714.1) was downloaded from NCBI GenBank (www.ncbi.nlm.nih.gov). The sequencing results of COI of the current study were compared with its reference sequences, in which single-nucleotide polymorphism (SNP) positions were detected. Every identical sequence was considered as single haplotype. Multiple sequence alignment was conducted through ClustalW method in MEGAX software.^41^ A phylogenetic tree was constructed from haplotypes of CO1 gene via maximum likelihood method, The Kimura-2 parameter method was used to estimate genetic distance within CO1 gene of current study, with other related species using the same software.

## Results and discussion

### Effect of sex and age on body weight and morphometric measurements

The effect of sex on body weight (kg) and morphometric measurements (cm) in the study is depicted in Table 1. Body weight was observed significantly (*p* < 0.05) different between the sexes. The body weight of goats has been observed to be sexually dimorphic.^22^^,^^42–44^ In the present study, measurements were generally higher in males than females (Table 1). This is in consonance with Hamayun et al.^45^ Adeyinka and Mohammed,^42^ Nazeer and Shah^14^ and Akpa et al.^46^ On the other hand^47^ and Adamu et al.^48^ worked on West African Dwarf goats, Red Sokoto and Sahel goats and reported that females were superior in body weight and other morphometric parameters across all age groups, which is contradicting with the present study findings. The effect of sex on body weight and several morphometric features shows that there is a typical difference between sexes caused by hormonal action, with males developing quicker than females.^49^^,^^50^ The results also showed that sex had significant (*p*˂0.05) effect on the body weight, heart girth, body length and horn length and no significant (*p* > 0.05) effect on heights of wither and rump, head length, ear length, neck length and tail length in Surguli goats which is in consonance with the work of Aliyu et al.^51^ Effect of sex on body measurements has been reported in previous studies.^52^^,^^53^ The effect of age on body weight (kg) and morphometric measurements (cm) in the study is depicted in Table 2. Results of the present study showed that age had significant (*p* < 0.05) effect on height at rump, ear length, horn length and tail length and no significant (*p* > 0.05) effect on body weight, body length, heart girth, height at wither, head length and neck length (Table 2). The results are in agreement with the reports of Aliyu et al.^51^ Results of the present study revealed that body weight increased with the advancement of age and then declined after attaining optimum growth at the ages of 4 and 5 years respectively. These variations are in accordance with the early reports of Hamayun et al.^45^ and Fajemilehin and Salako.^47^ Similarly, Getahun et al.^54^ also reported that all body measurements increased as age group increased from 1.5 to 2 years and 3.5 to 4 years of age groups followed by a persistent decline.

**Table 1.** Effect of sex on the body weight (kg) and morphometric measurements (cm) of Surguli goats.

Parameters	Mean ± SE		*p*-Value
	Male	Female	
Body length	56.38^a^±0.70	53.45^b^±0.53	0.0026
Body weight	34.25^a^±0.49	32.21^b^±0.37	0.0028
Heart girth	81.06^a^±0.16	80.83^b^±0.12	0.0059
Height at wither	74.31 ± 1.25	70.63 ± 1.64	0.0858
Height at rump	77.09 ± 1.52	74.21 ± 1.15	0.1429
Head length	12.09 ± 0.83	10.73 ± 0.63	0.2093
Ear length	9.36 ± 1.23	8.47 ± 0.94	0.5723
Horn length	11.89^a^±0.48	8.00^b^±0.25	0.0092
Neck length	21.54 ± 0.63	22.05 ± 0.48	0.5324
Tail length	13.40 ± 0.46	13.37 ± 0.35	0.9713

Mean values in a row with different superscripts are different at (*p* < 0.05).

**Table 2.** Effect of age on the body weight (kg) and morphometric measurements (cm) of Surguli goats (mean ± SE).

Parameters	2 Year	3 Year	4 Year	5 Year	*p*-Value
Body length	54.25 ± 0.98	54.37 ± 1.38	54.51 ± 0.87	55.40 ± 0.98	0.6726
Body weight	32.92 ± 0.62	32.92 ± 0.62	33.45 ± 0.69	32.15 ± 0.98	0.7586
Heart girth	80.52 ± 0.19	80.77 ± 0.20	80.80 ± 0.22	80.85 ± 0.31	0.6307
Height at wither	70.62 ± 1.86	72.60 ± 1.66	72.62 ± 1.70	74.70 ± 2.63	0.0864
Height at rump	70.75^b^±1.50	74.90^a^±1.34	78.25^a^±1.80	79.25^a^±2.12	0.0046
Head length	11.00 ± 1.04	11.60 ± 0.93	11.65 ± 1.47	11.80 ± 1.53	0.9679
Ear length	7.37^b^±1.29	7.60^b^±1.15	9.25^ab^±1.29	13.75^a^±1.83	0.0383
Horn length	6.66^b^±0.31	7.07^b^±0.27	8.48^a^±0.31	8.87^a^±0.44	0.0002
Neck length	21.00 ± 0.74	21.80 ± 0.66	22.25 ± 0.84	23.00 ± 1.05	0.4397
Tail length	12.80^c^±0.47	13.00^b^±0.42	13.50^ab^±0.47	15.30^a^±0.66	0.0284

Mean values in a row with different superscripts are different at (*p* < 0.05).

All measurements for different body parameters were included in model and stepwise elimination process was assumed to exclude the unfit body measurements. Results of the presents study showed that body weight of Surguli goat was predicted more precisely using all the body measurements in the present study, however, due to higher adjusted *R*^2^ value of 92%, suggested that the employed model was best fitted (Table 3). Many researchers found the influential role on the regression model of body weight to all body measurements at all ages and sex.^55^ The results of the current study are also supported by the study of Iqbal et al.^56^ who worked on prediction of body weight through body measurements in Beetal goats.

**Table 3.** Equations for predicting live weight of Surguli goat from body parameters.

Prediction equation	Adj. *R*^2^ (%)	*p*-Value	SE		Ind. variable
			b_0_	b_1_	
−3.95 + 0.67x	92	0.0001	1.11	0.02	Body length
−132.4 + 2.05x	44	0.0001	19.35	0.23	Hearth girth

Adj. *R*^2^ (%) = adjusted *R*^2^ (%); SE: standard error; Ind. variable: independent variable.

### Polymorphism and genetic diversity in the Surguli goat population

Analysis of amplified region (767-bp) of *CO1* gene identified polymorphic sites at the positions 5, 8, 10, 12, 13, 33, 260, 350 and 756, which might be used as genetic markers amongst individual animals within population of the Surguli goat as shown in Table 4.

**Table 4.** SNPs positions in nucleotides sequence of CO1 gene with reference sequence *Capra hircus* mitochondrial genome accession no (KP662714.1).

Polymorphic sites										
		5	8	10	12	13	33	260	350	756
1	**KP662714**	**G**	**T**	**G**	**T**	**A**	**C**	**C**	**T**	**G**
2	M33	.	.	T	.	.	.	.	C	.
3	M34	.	.	T	.	G	T	.	.	.
4	M35	.	.	.	.	.	T	.	.	.
5	M36	.	.	.	.	.	T	.	.	.
6	M37	.	C	.	.	.	T	T	.	.
7	M38	A	.	.	.	.	T	.	.	A
8	M39	.	C	T	.	.	T	.	C	.
9	M40	.	.	.	.	.	T	.	C	.
10	M41	.	.	.	G	.	T	.	.	.
11	M42	.	C	T	.	.	.	.	.	.

M33…M42: tag no of surguli goat samples in which SNPs were identified; A: adenine; C: cytosine; G: guanine; T: thymine.

Eight haplotypes were identified based on polymorphic sites found in nucleotide sequences of the *CO1* gene when compared to the mitochondrial genome of the Capra *hircus*. Every identical polymorphic site in *CO1* gene sequence was assumed to be the same haplotype. The largest haplotype comprised of two individuals whereas the remaining 7 haplotypes were represented by single sequence as data files obtained through DnaSP6 Software. The dots indicating identical base pairs with the reference mitochondrial genome of the Capra *hircus* as presented in Table 4. Both transversion and transition cause changes in nucleotide composition, which could be employed as genetic markers to study natural populations.^29^^,^^57^^,^^58^ Nucleotide substitutions caused by transversion, or transition can display a detailed picture of a population’s evolutionary and geographic history.^57^^,^^58^ The nine polymorphic locations were determined by comparing the *COI* gene sequences of Surguli goat samples with GenBank sequences of other goats, which revealed that the *CO1* gene has low polymorphism. In a previous study on *CO1*, similar low polymorphism was reported in Indonesian Lakor goat sequences having four polymorphic positions.^59^ When compared to previous investigations on other mitochondrial regions, the *CO1* gene showed low polymorphism as^60^ have obtained only four polymorphic sites in *CO1* sequences of Azi-kheli River buffaloes as compared to mt D-loop region where 28 polymorphic sites were identified. Similarly, Sultana et al.^61^ also reported 129 polymorphic sites in Pakistani domestic goats by using mtDNA D-loop and cytochrome b gene sequences.

The mean intra-specific diversity of nucleotide sequences of CO1 within Surguli goats as well as the mean inter-specific diversity of present study with closely related goats’ species were calculated by Kimura-2 parameter method in MEGAX software^41^ as shown in Table 5. The published nucleotide sequences of CO1 related goat breeds were assembled from NCBI data base in the current study included *Capra Ibex* COX1 gene (NC_020623.1); Meigu goat (*C. hircus*) (KM244714.1); Chinese Tibetan goat (*C. hircus*) (KJ940969.1); Qaidam Cashmere goat (*C. hircus*) (MG603753.1). The nucleotide sequence of the CO1 gene may be used to successfully identify species, for which the sequence variation should be sufficiently low within species while the variation among closely related species needs to be sufficiently high that a clear threshold between interspecific and intraspecific genetic variations can be demarcated.^62^ The results of present study showed intraspecific diversity of 0.23% within Surguli goats and interspecific diversity of 2.36% with other related species. Based on previous studies, 2.5% sequence difference is recommended in the CO1 gene sequence for species identification,^63^ while another study suggested that higher than 2 percent CO1 nucleotide sequence variation is enough to distinguish animal species.^64^ The outcomes of present study for distinction of Surguli goat with closely related goats species is in consonance with previously reported studies.

**Table 5.** Intraspecific diversity within CO1 sequences of the samples (M33-M42) and interspecific diversity with CO1 sequences of closely related species with specific accession numbers assembled from NCBI.

	1	2	3	4	5	6	7	8	9	10	11	12	13	14
KP662714														
M33	0.0025													
M34	0.0038	0.0038												
M35	0.0012	0.0038	0.0025											
M36	0.0012	0.0038	0.0025	0.0000										
M37	0.0038	0.0063	0.0050	0.0025	0.0025									
M38	0.0038	0.0063	0.0050	0.0025	0.0025	0.0050								
M39	0.0050	0.0025	0.0038	0.0038	0.0038	0.0038	0.0063							
M40	0.0025	0.0025	0.0038	0.0012	0.0012	0.0038	0.0038	0.0025						
M41	0.0025	0.0050	0.0038	0.0012	0.0012	0.0038	0.0038	0.0050	0.0025					
M42	0.0025	0.0025	0.0038	0.0038	0.0038	0.0038	0.0063	0.0025	0.0050	0.0050				
MG603753.Qaidem	0.0014	0.0028	0.0014	0.0014	0.0014	0.0028	0.0028	0.0028	0.0028	0.0014	0.0014			
KJ940969.tibetan	0.0014	0.0000	0.0014	0.0014	0.0014	0.0028	0.0028	0.0000	0.0000	0.0014	0.0014	0.0019		
KM244714.Meigu	0.0014	0.0000	0.0014	0.0014	0.0014	0.0028	0.0028	0.0000	0.0000	0.0014	0.0014	0.0019	0.0000	
NC_020623. Capra_Ibex	0.0295	0.0281	0.0295	0.0295	0.0295	0.0309	0.0309	0.0281	0.0281	0.0295	0.0295	0.0310	0.0304	0.0304

### Molecular phylogeny of the Surguli goat through COI sequences

**Figure 2.**
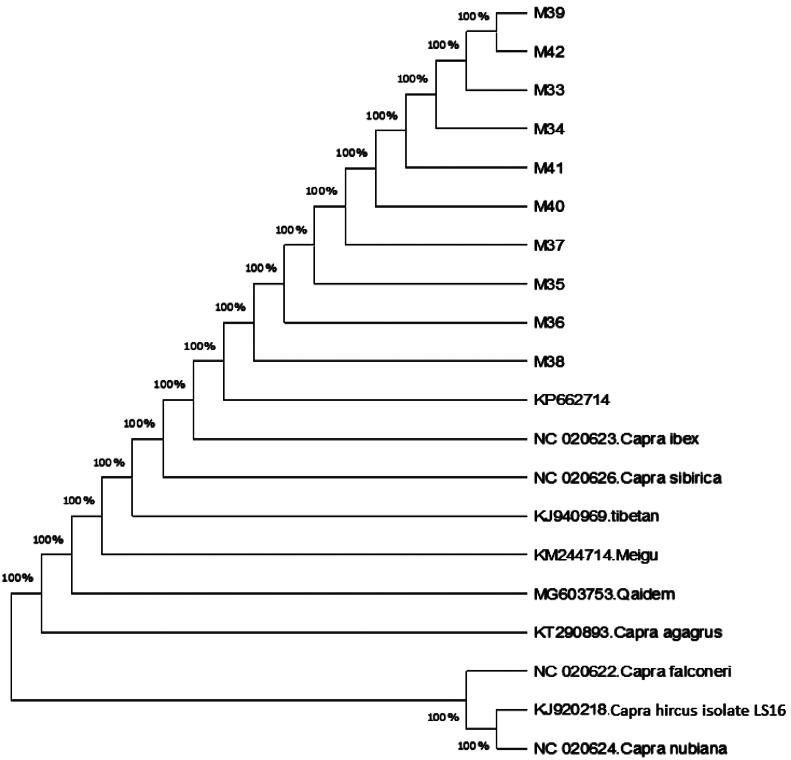
Phylogenetic tree constructed by N–J method and Tamura-Nei model in MEGAX software from CO1 haplotypes found in the present study and previously published haplotypes of other related species assembled from NCBI.

Phylogenetic relationship of the Surguli goat samples within nucleotide range 1–767-bp of the COI and other published *C. hircus* species sequences retrieved from GenBank was performed. Phylogenetic tree was built from the haplotypes of CO1 nucleotides sequence with one reference haplotypes of *C. hircus* mitochondrion (KP662714.1) along with already reported haplotypes of closely related capra species including *C. Ibex* COX1 gene (NC_020623.1); *C. Falconeri* COX1 gene (NC_020622.1); *C. Sibirica* COX1 gene (NC_020626.1); Meigu goat (C. hircus) (KM244714.1); Chinese Tibetan goat (*C. hircus*) (KJ940969.1); Qaidam Cashmere goat (*C. hircus*) (MG603753.1); *Capra nubiana* COX1 gene (NC_020624.1); *C. Aegagrus* mitochondrion (KT290893.1); *Capra hircus* Isolate LS16 (J920218.1), through Neighbor-Joining and Tamura-Nei model toolkits in MEGAX software. The evolutionary paradigm displayed that the CO1 gene may clearly distinguish the intra and inter-species levels linked to phylogenetic tree. The phylogram branches built on the COI gene sequences exhibited that Surguli goat is grouping with others individuals that were used to design this phylogenetic tree as shown in the Fig. 2. The present study phylogenetic tree revealed that evolutionarily, Surguli goat built-up a close relationship with wild goats’ species; *C. Ibex* COX1 gene (NC_020623.1), *C. Aegagrus* mitochondrion (KT290893.1), Qaidam Cashmere goat (*C. hircus*) (MG603753.1), *C. Sibirica* COX1 gene (NC_020626.1) and with two Chinese goat breeds; The Meigu goat (KM 244714.1), Chinese Tibetan goat (*C. hircus*) (KJ940969.1).

A well-resolved phylogenetic tree can help to define the species’ taxonomy, population history and evolutionary processes.^59^ The COI gene has successfully proven to distinguish between various ruminant species and could be consider as an efficient tool for the ruminants’ identification and their classification into different taxonomic categories.^65^ Ali et al.^36^ also suggested that COI gene sequences can be used as a possible tool to differentiate local Pakistani goat breeds from the exotic ones.

The phylogenetic tree revealed that the Surguli goats shared ancestry tree with *C. ibex* COX1 gene (NC_020623.1), *C. aegagrus* mitochondrion (KT290893.1), Qaidam Cashmere goat (*C. hircus*) (MG603753.1), *C. sibirica* COX1 gene (NC_020626.1) and with two Chinese goat breeds: The Meigu goat (KM 244714.1), Chinese Tibetan goat (*C. hircus*) (KJ940969.1). Mason^66^ has reported *C. aegagrus* (bezoar) as the direct ancestor of the domesticated goat and *C. falconeri* (Markhor) as the contributor to mostly Asian goats. According to previous studies *C. aegagrus* was considered as a single ancestor to Asian domestic goats.^67^^,^^68^ The alternative hypothesis by Harris^69^ proposed that at least two other species of wild goats, the *C. falconeri* (Markhor) and the Ibex (*C. ibex*) are possible candidates as ancestors for the domestic goats. In addition, Chen et al.^70^ also reported that the Asian domestic goats are originated and evolved from two wild types of goats (*C. aegagrus* and *C. ibex*) of Middle East, which are still existing in Tibet and Inner Mongolia. The goats are possibly domesticated about 10,500 years ago in high, rocky mountainous areas spreading from the Taurus mountains of Turkey into Pakistan^71^ and then spread swiftly because of human migration and trade patterns.^72^^,^^73^ This hypothesis supports our results, which showed that *C. ibex* might be the patriarchic descendant of the Surguli goat studied in this research, which has gone through adaptation in various environment at different periods.

The Neighbor-Joining tree topology (Fig. 2) clearly showed that Surguli goat and other goats from GenBank have close evolutionary relationship. According to Rumanta et al.^59^ the three wild species of goat; Ibex (*Capra ibex*), Bezoars (*Capra aegagrus*) and Markhors (*Capra falconeri*) have close relationship to the present-day domestic goat (*C. hircus*). However, the two wild species were ruled out, because the present study results obtained by phylogenetic analysis of CO1 clearly showed that these species are distantly related to Surguli goat. Moreover, the present study results are also supported by Khan et al.^12^ who reported, Markhor (*Capra falconeri*) and Ibex (*Capra ibex*) as the wild relatives of the Pakistani goats. Similarly, Sultana et al.^61^ worked on D-loop and Cyt b regions sequences in Pakistani goats and proposed Sindh Ibex (*C.a. bylyti*) as a possible ancestor of domestic goats.

## Conclusions

Morphometry of Sex is a significant indicator of body weight, body length, heart girth and horn length while age of the animals could be used as best predictor of rump height, ear length, horn length and tail length. This could be exploited as a selection tool for breeding and overall improvement. The molecular characterization indicated that Surguli goat have diverse population structure having high genetic diversity which can be used for strategic selection and further multiplication. The Neighbor Joining phylogenetic tree revealed that Capra *Ibex* and native Surguli goat have common ancestors.

## Supplementary Material

Supplemental Material
