# Supplementary material for: Morpometric and molecular characterization of Surguli goat through CO1 gene in district Kohat
Source: Anim Biotechnol. 2023 Dec 23;35(1):2290528. doi: 10.1080/10495398.2023.2290528 (PMC12674387; doi:10.1080/10495398.2023.2290528)
Supplement: Supplemental Material [file LABT_A_2290528_SM1079.docx]

# ANNEX 1

**INDIVIDUAL GOAT’s OBSERVATION AND MEASUREMENT PROFORMA FOR SURGULI GOAT**

**Flock no: Owner name: Location:**

| **Main parameters** | **Sub-parameters (cm)** | **Observation** |
| --- | --- | --- |
| Ear tag No & Sex: |  |  |
| Age (Months) | Reported |  |
|  | Detention |  |
| Head | Length |  |
|  | Width |  |
| Body | Length |  |
| Neck | Length |  |
| Body weight (Kg) |  |  |
| Girth | Heart |  |
|  | Belly |  |
| Body height | At Whiter |  |
|  | At Rump |  |
| Ears | Present/Absent |  |
|  | Ears length |  |
|  | Ears width |  |
| Horns | Length |  |
|  |  |  |
|  | Circumference |  |
